# A comparative analysis of proteins that accumulate during the initial stage of root hair development in barley root hair mutants and their parent varieties

**DOI:** 10.1007/s13353-012-0105-1

**Published:** 2012-07-31

**Authors:** Agnieszka Janiak, Stanisław Piórko, Andrea Matros, Hans-Peter Mock, Mirosław Kwaśniewski, Karolina Chwiałkowska, Beata Chmielewska, Iwona Szarejko

**Affiliations:** 1 Department of Genetics, University of Silesia, Jagiellońska 28, 40-032 Katowice, Poland; 2 Leibniz Institute of Plant Genetics and Crop Plant Research (IPK), Gatersleben, Germany

**Keywords:** Barley, Mutants, Root hairs, Root proteomics

## Abstract

The mechanisms of root hair formation have been studied extensively in *Arabidopsis* but knowledge about these processes in monocot species is still limited, especially in relation to the proteome level. The aim of this study was to identify the proteins that are involved in the initiation and the early stage of root hair tip growth in barley using two-dimensional (2D) electrophoresis and mass spectrometry. A comparison of proteins that accumulate differentially in two root hair mutants and their respective parent varieties resulted in the identification of 13 proteins that take part in several processes related to the root hair morphogenesis, such as the control of vesicular trafficking, ROS signalling and homeostasis, signal transduction by phospholipids metabolism and ATP synthesis. Among the identified proteins, two ATP synthases, two ABC transporters, a small GTPase from the SAR1 family, a PDI-like protein, a monodehydroascorbate reductase, a C2 domain-containing protein and a Wali7 domain-containing protein were found. This study is the first report on the proteins identified in the initial stage of root hair formation in barley and gives new insights into the mechanisms of root hair morphogenesis in a monocot species.

**Electronic supplementary material:**

The online version of this article (doi:10.1007/s13353-012-0105-1) contains supplementary material, which is available to authorized users.

## Introduction

Root hairs are a specific type of plant cells that are characterised by tip growth. This type of growth results in the formation of tubular-shaped extensions of root epidermal cells, which enlarge the root surface considerably. It has been shown that root hairs take part in the uptake of most macro- and micro-nutrients and that they are especially important for the uptake of phosphorous and iron. The amount of phosphorus uptake is positively correlated with the length and density of root hairs, which makes them an important structure for the utilisation of nutrients in low-phosphorus soil (Gilroy and Jones 2000). There are contradictory reports on the role of root hairs in overall plant development and yield, ranging from no effect to severe nutrient deficiency syndromes (Wen and Schnable 1994; Gahoonia and Nielsen 2004), and the particular response seems to be genotype-dependent. However, the specific way of root hair formation, which leads to the effective exploitation of the soil, makes root hairs an excellent model to study the molecular mechanisms of this highly organised type of growth.

Mechanisms of root hair development have been studied extensively in *Arabidopsis thaliana* (for a review, see: Carol and Dolan 2006; Libault et al. 2010). The process has been conventionally divided into several stages: patterning of the root epidermis, initiation of the root hair and formation of the bulge, transition to tip growth and the tip growth itself. The patterning of root hairs in *Arabidopsis* is under the control of several transcription factors and hormone signalling (Schiefelbein et al. 2009). During the initiation of root hairs, the Rop2 GTPase, a member of Rho-related GTPase from plants, appears at the initiation site and controls the Ca^2+^ gradient formation and F-actin accumulation at the bulge site. The pH at the initiation site drops, which probably activates cell expansines that are responsible for loosening the cell wall. The initiation of root hairs also relies on ethylene and auxin signalling. The transition to tip growth and root hair elongation relies mostly on the formation of the cytoskeleton and its rearrangements, vesicle transportation and the secretion of cell wall compounds, which is controlled by cellular signalling and the activity of various kinases, GTPases and other secondary agents (Guimil and Dunand 2007).

The availability of information on the molecular aspects of root hair development in monocot species is limited to some studies on maize (Wen et al. 2005; Hochholdinger et al. 2008), rice (Yuo et al. 2009) and barley (Kwasniewski and Szarejko 2006), which concentrated on the identification of single gene functions that were studied in mutants. Additionally, there are data on transcriptome analysis of selected root hair mutants in barley via microarray hybridisation (Kwasniewski et al. 2010). The proteomic approach to studying root hair morphogenesis has not yet been applied, for either *Arabidopsis* or barley. Proteomic experiments are limited to the studies conducted for soybean and maize, for which protein reference maps from isolated root hair cells were developed (Brechenmacher et al. 2009; Nestler et al. 2011), and to the study of the process of nodulation after the infection of soybean root hairs by *Bradyrhizobium japonicum* (Wan et al. 2005).

The collection of barley root hair mutants that was created and investigated in our laboratory (Szarejko et al. 2005) provides an opportunity to analyse the mechanisms of root hair formation at different stages of their development. The comparison of mutants and their parent varieties has the advantage of working in a system of the same genetic background where the studied genotypes differ in only a very few loci and all differences in gene expression and the subsequent synthesis of different proteins result, with a very high probability, from a mutation leading to an altered phenotype. In this study, the results of experiments performed using 2D electrophoresis and mass spectrometry, which were aimed at identifying proteins differentially accumulated in root hair mutants and their parent varieties during early stages of root hair morphogenesis in barley, are presented.

## Materials and methods

### Plant material and growth conditions

**Fig. 1:**
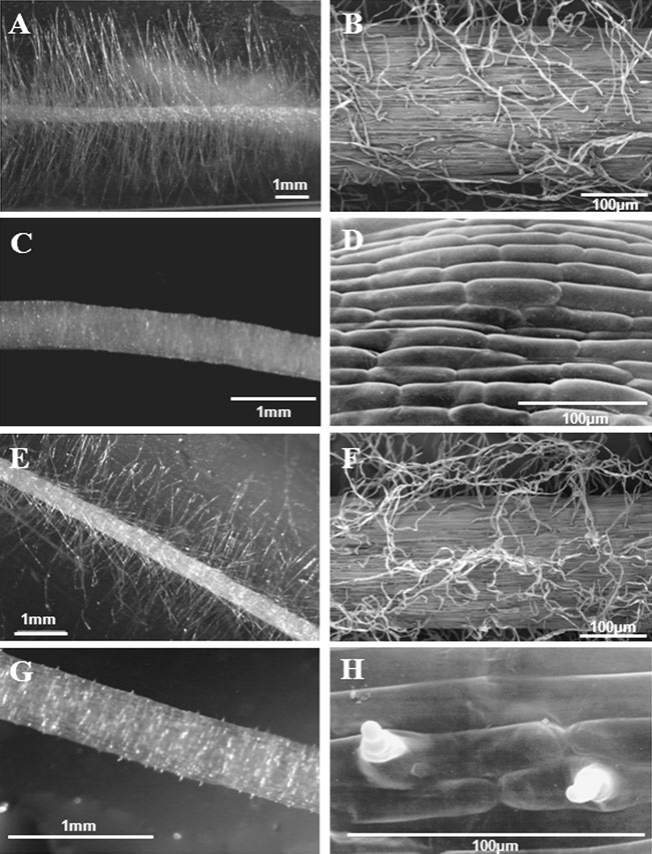
Root hair images of mutants and their parental varieties: **a**, **b** ‘Karat’ variety; **c**, **d** *rhl1.a* mutant; **e**, **f** ‘Dema’ variety; **g**, **h** *rhp1.b* mutant. **a**, **c**, **e**, **g** Photos from a stereoscopy microscope. **b**, **d**, **f**, **h** Photos from a scanning microscope

The seedlings of two root hair mutants, *rhl1.a* root hairless mutant and *rhp1.b* mutant with root hairs stopped after the formation of the bulge, together with their respective parent varieties, ‘Karat’ and ‘Dema’, were used as the material (Fig. 1). The mutant *rhl1.a* was obtained after chemical treatment of the ‘Karat’ variety with N-methyl-N-nitrosourea (MNU) and the mutant *rhp1.b* after a double treatment of the variety ‘Dema’ with sodium azide and MNU. The root hair phenotype of both mutants was monogenic and recessive. To clean the mutant background from other mutations, the mutant lines from M_10_–M_12_ generations were crossed with respective parent varieties. Then, F_2_ plants which showed a mutant phenotype were backcrossed again with the appropriate parent variety and the recombinants from the subsequent F_2_ generation that showed a mutate root hair phenotype were used for proteomic studies.

Seeds were sterilised in 20 % ACE detergent for 20 min, washed three times in sterile water and transferred to a Petri dish filled with wet vermiculite. The seeds were left at 4 °C overnight and then transferred to a growth chamber at a temperature of 22 °C ± 1 °C for 24 h in order to start germination. Afterwards, an aeroponic culture was prepared. The germinated seeds were placed in sterile glass tubes with cotton bungs. Each tube with seed was connected to an empty tube. Both parts were stuck together with parafilm and the bottom parts were protected from the light. The seedlings were grown in the growth chamber (light intensity 180 μEm^−2^ s^−1^, photoperiod 16 h and 22/18 °C ± 1 °C, day and night temperatures, respectively) for 5 days.

### Protein extraction

Roots from 15 seedlings per biological replication were collected for protein extraction. Three replications were used. Proteins were extracted using the TCA precipitation method. Briefly, the material was ground in liquid nitrogen and proteins were precipitated in ten volumes of 10 % (w/v) TCA in acetone with 20 mM DTT for 1 h at −20 °C. After 30 min of centrifugation (10 000 × g, 4 °C), the pellet was washed twice in acetone with 20 mM DTT, followed by centrifugation at the same parameters. The pellet was air-dried and dissolved in a lysis buffer (7 M urea, 2 M thiourea, 2 % w/v CHAPS, 0.002 % bromophenol blue, a 0.5 % IPG-buffer, 1.2 % v/v of destreak reagent, GE Healthcare, Piscataway, NJ, USA). The protein concentration was measured using a 2D quant kit with BSA protein as a standard (GE Healthcare, Piscataway, NJ, USA).

### Two-dimensional electrophoresis

The electrophoresis in the first dimension was carried out using 24-cm immobilised pH-gradient IPG strips with a range of 4–7 pH (GE Healthcare, Piscataway, NJ, USA). The IPG strips were rehydrated with 250 μg of total proteins for 12 h, first at 30 V (6 h) and later at 60 V (6 h). IEF was carried out on a IPGphor II (GE Healthcare, Piscataway, NJ, USA) at 20 °C with a current limit of 50 μA per strip under the following conditions: 200 V, 1 h; 500 V, 1 h; 1,000 V, 30 min; 8,000 V, 30 min (gradient); and 8,000 V, up to 12 h.

After IEF, the gel strips were equilibrated for 15 min in a 10-ml equilibration solution (6 M urea, 30 % glycerol, 2 % SDS, 0.002 % bromophenol blue, 50 mM Tris pH 8.8) containing 100 mg ml^−1^ DTT and then for 15 min in a 10-ml equilibration buffer containing 250 mg ml^−1^ iodoacetamide. The second dimension was carried out on an Ettan DALTsix system (GE Healthcare, Piscataway, NJ, USA) with self-casted 12 % SDS polyacrylamide gels. The electrophoresis conditions were as follows: 15 °C, 2 W per gel for 1 h and 17 W per gel for 4 h.

After electrophoresis, gels were fixed for 30 min in a fixing solution (30 % ethanol, 2 % phosphoric acid), double washed in 2 % phosphoric acid for 10 min and stained overnight in colloidal Coomassie Brilliant Blue staining solution (20 % ethanol, 10 % phosphoric acid, 10 % w/v ammonium sulphate, 0.12 % w/v Coomassie Brilliant Blue G-250). Excess of Coomassie Brilliant Blue staining was removed using Milli-Q water. Gels were scanned in the transmissive mode to obtain 16-bit TIF images that were used for the analysis with Dymension 2.0 software (Syngene, Cambridge, UK). Spot detection was automatically performed using the default settings of the software and manually edited in the case of detection errors. Gel normalisation was based on the total spot volume and normalised values were used for spot quantification. Normalised spot volumes were then compared between the samples using the two sample *t*-test with the assumption of unequal variances.

### Protein identification via mass spectrometry

Spots selected for the identification were manually excised from the gels—for each spot, the excision was done in at least two replicates. The spots were washed in 100 μl 100 mM acetonitrile:50 mM ammonium bicarbonate (50:50, v/v) for 30 min at room temperature with occasional mixing, incubated for 20 min in 100 % acetonitrile and completely air dried. Dried spots were rehydrated with 7.5 μl of a trypsin solution [75 ng of trypsin, Promega, Madison, WI, USA, in 5 mM ammonium bicarbonate and 5 % acetonitrile (v/v) buffer per spot] and incubated for 5 h at 37 °C. The digestion was stopped by the addition of 1 μl 1 % trifluoroacetic acid (TFA).

An aliquot of 2–4 μl of protein digest was separated on a nanoACQUITY system (Waters, Milford, MA, USA) equipped with a 20 mm × 180-μm Symmetry (5 μm) pre-column coupled to a 100 mm × 100-μm BEH (1.7 μm) C18 analytical column, with a gradient of 3–40 % acetonitrile over 30 min at a constant flow rate of 600 nl min^−1^. Solvent A consisted of water with 0.1 % formic acid and solvent B of acetonitrile with 0.1 % formic acid. The lockmass, [Glu1]-Fibrinopeptide B human (Sigma-Aldrich, Seelze, Germany) at a concentration of 500 fmol μl^−1^, was delivered at a constant flow rate of 600 nl min^−1^ through the auxiliary pump of the nanoACQUITY system to the reference sprayer of the NanoLockSpray source. Mass spectrometric detection was done on a Q-TOF Premier mass spectrometer (Waters, Milford MA, USA) in a data-dependent analysis (DDA) mode using following conditions: MS was operating in v-mode and positive nanoelectrospray ion mode. The applied source temperature was 80 °C and cone gas flow 50 l h^−1^. Mass spectra for the calibrant were acquired in the continuous fragmentation mode at a collision energy of 22 eV. MS data were acquired in the m/z range of 400–1,600, with a survey scan time of 0.95 s and an interscan time of 0.05 s. MS/MS was performed in the m/z range of 50–1,600 when the total ion chromatogram (TIC) intensity was above a threshold of 2,000.

Protein Lynx Global Server (PLGS) 2.3 software (Waters,, Milford MA, USA) was used for the processing and identification of obtained spectra. Database searches were conducted against the barley EST sequences of the HarvEST database, EST Gene Index of the TIGR database for the *Poaceae* family (release: October 2010) and UniProt_90 reference database for *Viridiplantae* (release: 2010/07/26). The search parameters were: 10 ppm mass tolerance, 0.1 Da fragment mass tolerance, one missed cleavage site, oxidation (Met), propionamide (Cys) and carbamidomethyl (Cys) as variable modifications. Protein identification was accepted when at least two peptide fragments per protein were identified and a probability score higher than 90 % was obtained (PLGS score > 12). In some cases, a PLGS score < 12 was accepted; this occurred for proteins related to redundant entries in the database. In addition, a spot’s identity was confirmed on at least two separate gels. BLAST homology and similarity-based searches were performed with the same databases. The sequences of the proteins identified were additionally examined using the Conserved Domain Database (CDD) available on the NCBI website (Marchler-Bauer et al. 2011). For the classification of proteins according to their biological function, data from UniProt (http://www.uniprot.org) and QuickGO Gene Ontology (http://www.ebi.ac.uk/QuickGO/) were used.

### Comparison of proteins that accumulate differentially with the expression of their corresponding genes

In our former study, which was aimed at a global transcriptome analysis of the same set of genotypes as presented here, it was possible to identify ten genes with a very strong reduction of expression in the root hairless mutant in comparison to its parent variety ‘Karat’ (Kwasniewski et al. 2010). The question arose as to whether among genes differentially expressed in transcriptome analysis there were the genes encoding proteins identified in the presented study as differentially accumulated. To answer this question, the corresponding probes on the GeneChip *Barley Genome* Array (Affymetrix) were identified and the expression level of the established genes in the roots of mutants and their parent varieties was evaluated using quantitative RT-PCR. First, the peptide sequences identified in this study were used to search for similar sequences in the GenBank non-redundant protein sequences database using the Blastp method. The first entries found for *Hordeum vulgare* within the top five Blastp results were treated as positive hits. All other entries for barley, with less significant e-values than the top five Blastp results, were treated as hits for presumable paralogues and were not considered in the further analysis. Corresponding cDNA/EST sequences were identified for the positive protein hits. Subsequently, the identified nucleotide sequences were used to search for matching probe sets on the GeneChip *Barley Genome* Array (Affymetrix) using the Blast tool from the PLEXdb database (http://www.plexdb.org; Dash et al. 2012). Next, a quantitative RT-PCR for the identified genes was performed. Primers were designed using the QuantPrime platform (http://www.quantprime.de; Arvidsson et al. 2008) based on the identified nucleotide sequences. Total RNA was isolated from 1-cm-long tip root fragments from the four genotypes used in the study: ‘Karat’, *rhl1.a* mutant, ‘Dema’ and *rhp1.b* mutant. Three replicates of each genotype were used. The dissected root fragments were homogenised in sterile mortars containing 550 μL of an RLT buffer (RNeasy Plant Mini Kit; Qiagen, Hilden, Germany). After homogenisation, total RNA was extracted using the RNeasy Plant Mini Kit according to the manufacturer’s instructions. Additional on-column digestion was carried out with DNAse I. The RNA was eluted by double elution in 35 μL of sterile, RNase-free water. The yield and purity of RNA was determined using a NanoDrop ND-1000 spectrophotometer (NanoDrop Technologies, Wilmington, DE, USA). A single-stranded cDNA synthesis was performed from 1 μg of the total RNA in 20-μL reactions using a Maxima First Strand cDNA Synthesis Kit for RT-qPCR (Fermentas/Thermo Fisher Scientific). The obtained cDNA was diluted 1:5 with ddH_2_O and used as a template for the quantitative PCR. The reference gene *RPII* (RNA polymerase II largest subunit; GenBank: NIASHv2125A18) was used in the study. The following qPCR protocol was used on the LightCycler 480 Real-Time PCR Instrument (Roche) using the SYBR Green I method: initial denaturation for 10 min at 95 °C, followed by 10 s at 95 °C, 15 s at 56 °C and 10 s at 72 °C, and repeated in 45 cycles. At the end of the qPCR reaction, a melting curve analysis was performed. qPCR was performed in technical duplicates for each of the three biological replicates. Amplification efficiencies were calculated with the LinRegPCR tool (Ramakers et al. 2003) using the best-fit method for 4 to 6 points. The differential expression of the analysed genes was calculated with the REST software (Pfaffl et al. 2002). The statistical significance of changes in gene expression between the wild-type plants (WT) and the corresponding mutants (*P* < 0.05) was calculated using the randomisation test implemented in the REST software.

## Results

**Fig. 2:**
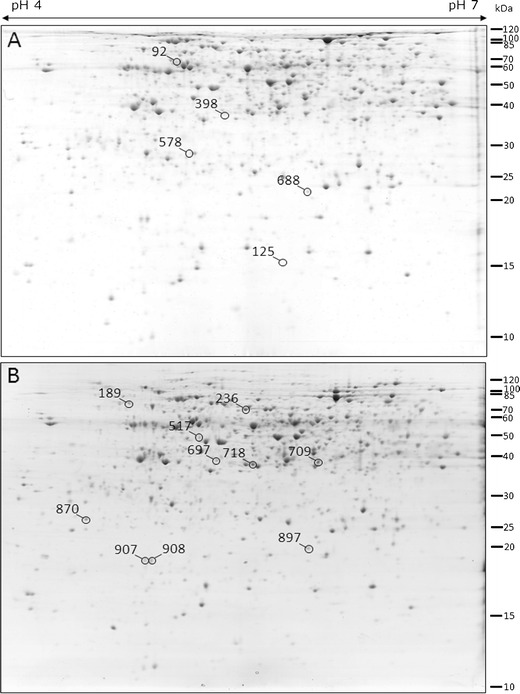
Protein spots that showed differences between root hair mutants and their parental varieties. **a** 2D gel image of the ‘Karat’ variety root proteome; **b** 2D gel image of the ‘Dema’ variety root proteome (In order to ensure high enough quality in printing, the contrast was slightly enhanced in an identical manner for both images)

**Fig. 3:**
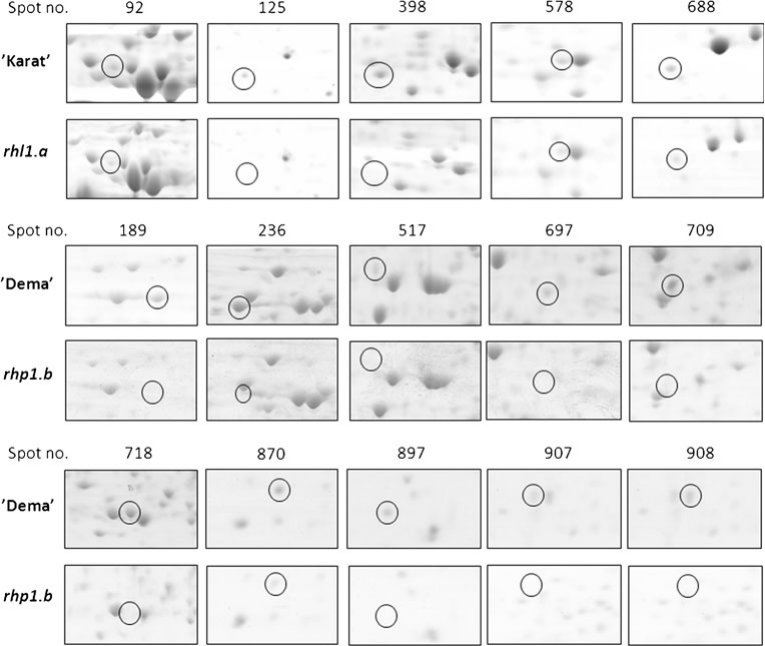
Close-up images of spots of differing parent varieties (‘Karat’ and ‘Dema’) and their respective root hair mutants (*rhl1.a* and *rhp1.b*). The figure represents single, raw images of spots

Two pairs of barley genotypes were compared in the presented study: root hairless mutant *rhl1.a* with its parent variety ‘Karat’, and the mutant *rhp1.b* with root hairs stopped after the formation of the bulge with its parent variety ‘Dema’. After the 2D separation of proteins extracted from roots and gel image analysis of three biological replications, 659 and 670 consensus spots were obtained for *rhl1.a* ‘Karat’ and *rhp1.b* ‘Dema’, respectively. In general, the 2D gel images from each pair of the compared genotypes showed a very high similarity and a detailed comparison resulted in the identification of five spots that were differentially accumulated in the ‘Karat’ variety in relation to *rhl1.a* and ten spots that differentiated the ‘Dema’ variety and the *rhp1.b* mutant (Figs. 2 and 3). In the first group of five spots, one was present only in ‘Karat’ and the remaining four were more abundant in ‘Karat’, with the fold change ranging from 2.04 to 5.44 over the spot volumes observed in the gels from the *rhl1.a* plants. In the second group of ten spots, seven were present only in ‘Dema’ and three were more abundant in ‘Dema’ than in the *rhp1.b* plants, with the fold change ranging from 1.83 to 9.64. After subjecting the MS/MS spectra and peptide sequences to a database search, a total of 13 spots were identified (Table 1). The number of peptides that allowed the identification of a protein varied from 2 to 18, with an average of 6.5 peptides per spot. Similarly, the coverage of protein sequences varied from 5.98 to 32.62 % (Supplementary Table 1, available online). As the identification of proteins via MS/MS was made in replicates, the proteins were considered to be positively identified only if both replicates gave the same hit to the database entry.

**Table 1:** Proteins differentially expressed between parent varieties and their respective root hair mutants identified using 2D gel image analysis and mass spectrometry

Spot	Matching protein	Accession no. (TIGR^a^, HarvEST^b^, UniRef90^c^)	P1/P2	E-pI	E-MW	T-pI	T-MW	Parent variety	Mutant	Fold change	*P*-value
								MNV (±SD)	MNV (±SD)		
‘Karat’ vs. *rhl1.a* mutant											
92	Vacuolar ATP synthase subunit B isoform 1	HV_TC204946^a^	7/9	5.23	65	5.12	54.02	0.0910 (±0.021)	0.0383 (±0.0261)	2.37	0.0277
125	na	na	na	5.92	15	na	na	0.0703 (±0.0135)	‘Karat’ only		
398	na	na	na	5.59	38	na	na	0.1686 (±0.0770)	0.031 (±0.0131)	5.44	0.0433
578	Aluminium-induced protein-like	HV_TC202901^a^	5/3	5.29	28	6.05	26.73	0.039 (±0.0092)	0.017 (±0.0056)	2.29	0.0163
688	GTP-binding protein SAR1A	HV_TC228420^a^	3/4	6.10	23	6.97	22.03	0.0906 (±0.0305)	0.044333 (±0.0232)	2.04	0.0546
‘Dema’ vs. *rhp1.b* mutant											
189	Protein disulphide isomerase (PDI)-like protein	35_1248^b^	7/9	4.84	75	4.80	63.35	0.067 (±0.0076)	‘Dema’ only		
236	2,3-bisphosphoglycerate-independent phosphoglycerate mutase	35_15021^b^	15/18	5.65	69	5.25	60.81	0.184 (±0.0199)	0.035 (±0.025)	5.26	0.0008
517	Monodehydroascorbate reductase	35_15199^b^	6/9	5.32	48	5.30	46.68	0.054 (±0.0064)	‘Dema’ only		
697	C2 domain-containing protein	Q5DVL6^c^	5/5	5.44	38	5.58	32.66	0.043 (±0.0124)	‘Dema’ only		
709	Malate dehydrogenase	35_169^b^	7/10	6.13	38	8.74	35.46	0.135 (±0.0253)	0.014 (±0.0029)	9.64	0.0068
718	Similar to UniRef100 Q0IZS5 Cluster Os09g0552600 protein (legumin-like protein)	35_18505^b^	6/6	5.68	37	8.52	33.13	0.156 (±0.0171)	‘Dema’ only		
870	PR17d precursor	35_414^b^	4/6	4.51	25	4.70	25.48	0.066 (±0.0279)	0.036 (±0.0102)	1.83	0.0980
897	ATP synthase subunit D mitochondrial	HV_BI953495^a^	7/9	6.04	20	5.09	19.58	0.053 (±0.0254)	‘Dema’ only		
907	ABC transporter-related	HV_TC195881^a^	1/2	4.96	18	9.03	28.61	0.056 (±0.0229)	‘Dema’ only		
908	ABC transporter-related	HV_TC195587^a^	4/2	5.02	19	9.03	28.61	0.104 (±0.0172)	‘Dema’ only		

P1, P2 - the number of MS peptides that matched in replicate 1 and 2 of spot identification via LC-MS/MS; E-pI – estimated experimental isoelectric point; E-MW – estimated experimental molecular weight (kDa); T-pI – theoretical isoelectric point; T-MW – theoretical molecular weight (kDa); MNV (±SD) – mean normalised spot volume calculated for three replicates of each sample ± standard deviation; Fold change – calculated for the relation:parent variety vs. mutant; na – protein not identified (no positive hits in the databases)

It was not possible to identify two protein spots differentiating the ‘Karat’ variety from the root hairless mutant *rhl1.a* because too few peptides matched the database entries. From among the spots that were positively identified, two matched the same protein sequence—the ABC transporter-related protein (spot nos. 907 and 908 from ‘Dema’). As these protein spots differ slightly in their estimated experimental pI and molecular weight, they might represent different isoforms of the same protein. Some discrepancies between the experimental and theoretical values of pI and molecular weight were also observed for most of the other protein identifications. The greatest difference between the experimental and theoretical pI was observed for spots 907 and 908, identified as ABC transporter-related proteins, for spot no. 718 that was similar to a protein similar to UniRef100 Q0IZS5 Cluster Os09g0552600 and for spot no. 709, which was identified as malate dehydrogenase. Some differences were also noticed for spot no. 92, representing subunit B of the vacuolar ATP synthase, spot no. 189, identified as PDI-like protein, and spot no. 236, identified as 2,3-bisphosphoglycerate-independent phosphoglycerate mutase. This might be explained by the fact that most of our identifications rely on barley EST sequences, which do not always represent the full protein sequence. In some cases, these differences might also result from post-translational modifications of the proteins that were analysed. It is also possible that proteins from the above-mentioned spots have the same domains as the proteins from the databases and represent the same protein families. They may, however, be novel members of the families indicated by the database search. The latter may be true for spots 907 and 908, where only a limited number of peptides were found to match the database entry.

Protein spots identified as differentially accumulated between mutants and their respective parent varieties were additionally compared between the ‘Karat’ and ‘Dema’ varieties. No statistically significant difference in the spot volumes was found for the majority of them (data not shown). Four spots, 236, 517, 709 and 718, differed significantly between the varieties, but the fold change merely exceeded the value of one, with the exception of spot no. 709, which was five times more abundant in the ‘Dema’ than in the ‘Karat’ variety. Despite some differences between varieties, the proteins identified as differing one mutant/parent variety pair were present on the 2D gels of the second mutant and its parent variety, and did not show differences in their accumulation between them. Two spots from the selected group were present in one variety only: spot no. 125, which failed to be identified by mass spectrometry, was present in the ‘Karat’, and spot no. 897, identified as the subunit D of mitochondrial ATP synthase, was present in the ‘Dema’ variety only.

The functions of the proteins identified encompass such processes as cellular transport, cell signalling, redox homeostasis, ATP synthesis, ATP catabolism, glucose metabolism, pathogen and stress response (Table 2). The majority of proteins identified in the presented study belong to several pathways that may be related to root hair development.

**Table 2:** Biological and molecular functions of the identified proteins

Spot	Protein name	Biological process	Molecular function
Proteins identified in the ‘Karat’ variety			
92	Vacuolar ATP synthase subunit B isoform 1	ATP synthesis coupled proton transport	Hydrogen ion transporting ATP synthase activity
578	Aluminium-induced protein-like	Response to stimulus	na
688	GTP-binding protein SAR1A *Arabidopsis thaliana*	Vesicle-mediated transport	GTP-binding
Proteins identified in the ‘Dema’ variety			
189	Protein disulphide isomerase (PDI)-like protein	Cell redox homeostasis	Oxidoreductase activity
517	Monodehydroascorbate reductase	Cell redox homeostasis	Oxidoreductase activity
697	C2 domain-containing protein	Signalling	Calcium/lipid-binding
236	2,3-bisphosphoglycerate-independent phosphoglycerate mutase	Glucose catabolic process	Manganese ion-binding; catalytic activity
709	Malate dehydrogenase	Glucose catabolic process	Oxidoreductase activity; catalytic activity
718	Similar to UniRef100 Q0IZS5 Cluster Os09g0552600 protein (legumin-like protein)	Unknown	Nutrient reservoir activity
870	PR17d precursor	Stress response	na
897	ATP synthase subunit D mitochondrial	ATP synthesis coupled proton transport	Hydrogen ion transmembrane transporter activity
907	ABC transporter-related	ATP transport	ATP binding
908	ABC transporter-related	ATP transport	ATP binding

na – not available

**Fig. 4:**
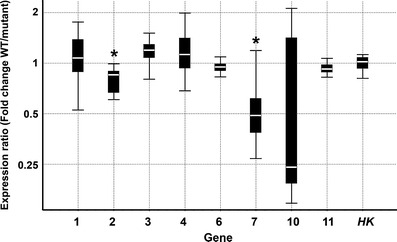
The expression analysis of genes representing the proteins identified in the study. The results are shown as a ratio of expression in the roots of the wild-types and the corresponding root hair mutants: Karat/*rhl1.a* (genes 1, 2 and 3) and Dema/*rhp1.b* (genes 4, 6, 7, 10 and 11). The *boxes* represent the middle 50 % of the observations, the *white line* represents the median gene expression and the *whiskers* represent the minimum and maximum observations. *Changes in gene expression between the wild-type plants (*WT*) and the corresponding mutants that are significantly different (*P* <0.05). *HK* housekeeping, reference gene *RPII*

A search for microarray probes corresponding to identified proteins using Blastp analysis against the non-redundant protein sequences database (GenBank) resulted in the identification of nine putative proteins encoded by corresponding cDNA/EST sequences (Table 3). None of the nine genes analysed was differentially expressed in the microarray experiment carried out in the corresponding system of Karat versus *rhl1.a* and Dema versus *rhp1.b* mutant (Kwasniewski et al. 2010). However, the qRT-PCR analysis revealed that two genes out of the eight genes tested were differentially expressed. A gene corresponding to an aluminium-induced protein-like protein (encoded as no. 2) was down-regulated in the roots of the root hairless mutant *rhl1.a* in comparison to the wild-type variety ‘Karat’ by a mean factor of 0.793 (*P* = 0.011). A gene corresponding to the C2 domain-containing protein (encoded as no.7) was down-regulated in the roots of the *rhp1.b* mutant in comparison to the wild-type variety ‘Dema’ by a mean factor of 0.511 (*P* = 0.014). The expression of all remaining genes except one did not differ between the roots of mutants and their respective parent varieties (in all cases, *P* > 0.05) (Fig. 4). It was not possible to reliably test the expression of one gene that corresponded to a legumin-like protein (encoded as no. 9) with regard to the lack of specificity of the qPCR reaction for this transcript.

**Table 3:** The results of Blastp analysis with the peptides identified in the presented study against the non-redundant protein sequences database (GenBank) and the corresponding probe sets on GeneChip *Barley Genome* Arrays (Affymetrix)

Spot no.	Code for protein/gene	GenBank accession (protein)	Blastp E-value	GenBank accession (nucleotide)	GeneChip *Barley Genome* Array probe set	Differential expression in microarray experiments^a^
92	1	Q40079	3e^−27^	AAA81331.1	Contig2464_at	–
578	2	BAJ94066	7e^−19^	AK362862	Contig4322_at	–
688	3	BAK00106	1e^−09^	AK368903.1	Contig3728_at	–
189	4	BAJ87919	7e^−13^	AK356704	Contig3884_at	–
236	5	Not identified	na	na	na	na
517	6	BAJ90391	1e^−26^	AK359180	Contig2943_s_at	–
697	7	CAI58613	2e^−11^	AJ630120	Contig112_at	–
709	8	Not identified	na	na	na	na
718	9	BAJ96477	1e^−22^	AK365274	Contig10263_at	–
870	10	CAA74593	1e^−21^	Y14201	Contig634_at	–
897	11	BAJ90886	7e^−10^	AK359677	Contig217_at	–
907	12	Not identified	na	na	na	na
908	13	Not identified	na	na	na	na

^a^The results of the inspection of microarray experiments carried out by Kwasniewski et al. (2010)

na – not available

## Discussion

In the presented study, the proteins that are differentially accumulated in the roots of barley root hair mutants and their parent varieties were identified. The mutated root hair characters were monogenic and recessive. The mutant lines used for the analysis were obtained by chemical mutagenesis and represent M_10_–M_12_ generations after mutagenic treatment. They were additionally cleaned of eventual other mutations by double backcrossing with their respective parent varieties. As the lines that were compared (mutants and parents) had the same genetic background, it can be assumed that the differences observed in protein accumulation were the effects of a mutation that caused a change in the root hair phenotype.

The lack of root hairs in the *rhl1.a* mutant might result from either a lack of a mechanism that leads to root hair initiation or a lack of establishment of the correct cell fate in epidermis cells. The phenotype of the second mutant used in the study, which developed slightly elongated root hair primordia, resulted from a defect in the transition to tip growth. The initiation and tip growth of root hairs, although functionally occurring one after the other, are most likely controlled by distinct molecular pathways.

One of the proteins differentially accumulated between ‘Karat’ and the root hairless mutant *rhl1.a* was the GTP-binding protein SAR1A (Secretion-Associated and Ras-related protein 1A). This protein is a member of the ARF (ADP-ribosylation factor) family of small GTPases that are involved in the process of vesicular trafficking, specifically, transport from the endoplasmic reticulum (ER) to the Golgi apparatus (Kim et al. 1997). Small GTPases have been reported to be involved in the process of root hair initiation in *Arabidopsis*, with the leading role being played by Rop2 GTPase, which is located at the site of the formation of the bulge and is continuously present at the tip of a growing root hair (Jones et al. 2002). The precise location of Rop2 is controlled by another GTPase factor, AFR1, which is present in the membranes of the Golgi apparatus and the endosomes. ARF1 is responsible for the apical-basal polarisation of the epidermal cell (Xu and Scheres 2005). The proteins from the SAR1 family have not previously been reported as being involved in root hair morphogenesis. Thus, the identification of the SAR1A protein as being differentially accumulated between the root hairless mutant and the wild-type root hair variety ‘Karat’ indicates the importance of this class of small GTPases in the process of root hair formation via the control of vesicular trafficking between the membranes of the ER and the Golgi apparatus.

Another candidate protein, an aluminium-induced protein-like protein, was identified as differentially accumulated between the ‘Karat’ variety and the root hairless mutant *rhl1.a*. Sequence analysis using CDD software (NCBI) revealed that this protein has a Wali7 domain that is characteristic for a protein of unknown function, which was identified in wheat after induction by aluminium (Richards et al. 1994). As the molecular function of proteins with the Wali7 domain is poorly understood, they may represent a novel group of factors synthesised not only in response to aluminium but which may also be responsible for some developmental processes in plant cells. It has been shown that aluminium treatment results in the activation of a broad range of processes in the cell: it changes the membrane potential, alters calcium homeostasis, disrupts the structure of the cytoskeleton in both microtubules and actin filaments, and increases the ROS levels in the cells (Panda et al. 2009). It generally affects the majority of the known mechanisms required for root hair development. Thus, it is possible that the proteins induced by aluminium treatment, in order to restore the cell homeostasis, would exhibit similarities to the factors directly involved in the developmental processes leading to root hair formation. Indirect evidence of such a possibility is the detection of genes encoding proteins with the Wali7 domain among the EST sequences from the root-hair enriched cDNA library of *Medicago truncatula* (Covitz et al. 1998) and among the proteins up-regulated in rice during the germination of pollen tubes, which are other types of cells characterised by tip growth (Dai et al. 2007).

Two enzymes involved in ATP synthesis were identified in the presented study: vacuolar ATP synthase (V-ATPase) differentially accumulated between the ‘Karat’ variety and the root hairless mutant *rhl1.a*, and the subunit D of mitochondrial ATP synthase present in the ‘Dema’ variety and lacking in the *rhp1.b* mutant. Additionally, the *rhp1.b* mutant lacked the ABC transporter-related proteins. The evidence that V-ATPase is involved in the regulation of cell expansion derives from the studies of *Arabidopsis vha-*c1 and *vha-*c3 mutants with a silenced expression of subunit c of the V_o_ subcomplex of the enzyme. Both mutants exhibited a decrease in root length. It has been suggested that the role of V-ATPase in cell expansion is to control protein trafficking and vesicle fusion in plants. The V-ATPase subunits are abundant in cells that are active in vesicular traffic and exocytosis, such as pollen tubes, where the secretion of cell wall materials is coupled with rapid tip growth (Padmanaban et al. 2004).

ATP is the most important source of energy for every reaction in the cell; however, if secreted outside the plasma membrane, it can serve as a signalling molecule (Roux and Steinebrunner 2007). It has been shown that extracellular ATP (eATP) plays a role in the polarised growth of root hairs through the increased production of ROS at the tip of the *Medicago truncatula* hairs. The application of eATP effectively promoted root hair growth (Kim et al. 2006). ATP can be transported outside of the plasma membrane by the ABC transporters (Weerasinghe et al. 2009) and members of this protein family were found to be present in the ‘Dema’ variety, whereas they were lacking in the *rhp1.b* mutant. It was reported that the perception of eATP results in an increase in the concentration of calcium ions in the cytosol and the production of superoxide (Roux and Steinebrunner 2007). The eATP seems to play a major role in the control of the cascade of processes in root hair tip growth.

Another group of proteins identified in this study is involved in the cell redox homeostasis. The lack of two such proteins, protein disulphide isomerase-like (PDI) protein and monodehydroascorbate reductase, was related to the inhibition of root hair growth shortly after the formation of the bulge. The analysis of the PDI-like protein using CDD software showed that it belongs to the thioredoxin (TRX)-like protein disulphide oxidoreductases that alter the redox state of numerous target proteins, including peroxidases and reductases (Nordberg and Arnér 2001), which is the one of the processes of cell signalling. The redox state of the root hair cell has been reported as an important mechanism involved in root hair morphogenesis in *Arabidopsis*. The mutation in the *rhd2* gene in *Arabidopsis*, which encodes the NADPH/RHD2 oxidase, leads to the formation of very short root hairs that are unable to continue their tip growth (Foreman et al. 2003). NADPH oxidase is responsible for the formation of the reactive oxygen species (ROS), which cause the activation of the calcium channels required for cell expansion during root hair growth (Foreman et al. 2003). The presence of ROS in the growing cell is an important signal but, at the same time, it results in the development of an antioxidant defence in order to prevent any oxidative damage to cell components. The PDI-like protein identified in our study may play a role in the ROS signalling pathways or in the response system to oxidative stress in root hair cells.

Such defence systems require substrates that can serve as antioxidants. The most abundant antioxidant is ascorbate, which acts as an ROS scavenger. Monodehydroascorbate reductase that was identified in the presented study is required for ascorbate regeneration from its oxidation products (Becana et al. 2010). A lack of this enzyme in the barley *rhp1.b* mutant may indicate that the process of ascorbate regeneration in the mutant is impaired.

Another protein present in the ‘Dema’ variety and lacking in the *rhp1.b* mutant was the C2 domain-containing protein. Such a domain is characteristic for one of the classes of plant phospholipases (PLDs). These enzymes are regulated by Ca^2+^ ions and are involved in cell signal transduction through the production of signal molecules after the cleavage of phospholipids (Zheng et al. 2002). Different PLDs have been reported to have an impact on the development of the tip-growing cells, including pollen tubes (Dowd et al. 2006) and root hairs (Schiefelbein et al. 2009). It has also been shown that both actin microfilaments and microtubules are regulated by PLDs, which, in turn, is associated with the regulation of vesicle transportation and membrane trafficking (Downes et al. 2005).

Among the proteins identified, malate dehydrogenase (MDH) and 2,3-bisphosphoglycerate-independent phosphoglycerate mutase were found after a comparison of the *rhp1.b* mutant and the ‘Dema’ variety. These proteins are involved in the primary metabolism of the cell and are probably not directly related to the process of root hair growth; however, they may play a role in supplying energy for developing outgrowth.

The role in root hair tip growth of the remaining two proteins identified in the presented study, the PR17d precursor present at a higher level in the plants with normal root hairs when compared to the *rhp1.b* mutant and the protein from spot no. 718 similar to UniRef100 Q0IZS5 Cluster Os09g0552600, which is present only in the ‘Dema’ variety, is unclear. The members of the PR17 protein family were first detected in barley after inoculation with *Blumeria graminis* f.sp. *hordei* and it has been speculated that they play a role in plant resistance response in relation to either cell wall metabolism or signal transduction (Christensen et al. 2002). Some of the PR proteins have been found to be accumulated during pollen development (Worrall et al. 1992), seed germination (Wu et al. 2001) and fruit development (Monteiro et al. 2007). Thus, the PR17d protein precursor may be involved the control of the developmental processes of the cell.

The protein similar to UniRef100 Q0IZS5 Cluster Os09g0552600 was found to have a cupin domain characteristic for storage proteins in plants that exhibit oxalate oxidase (OXO) activity or superoxide dismutase (SOD) activity (Zimmermann et al. 2006). Many cupin proteins are enzymes associated with cell wall synthesis, which is involved in cell wall strengthening (Dunwell et al. 2000). The identification of a protein with a cupin domain that was not present in the *rhp1.b* mutant might be associated with the last stage of root hair formation when the tip growth is already terminated and loosening of the cell wall is no longer required. It is likely that, in mutant plants that lack the mechanisms of root hair tip growth, there may also be a lack of the machinery that terminates root hair expansion.

The aim of our earlier studies was the identification of the genes involved in root hair development in barley and were concentrated on transcriptome profiling (Kwasniewski et al. 2010). The use of microarrays for transcriptome analysis between ‘Karat’ and *rhl1.a* root hairless mutant led to the identification of ten differentially expressed genes, including the genes for peroxidases, xyloglucan endotransglycosylase (XET), an arabinogalactan protein (AGP), extensin, a leucine-rich-repeat (LRR) protein, a Rho GTPase GDP dissociation inhibitor (RhoGDI) and a phosphatidylinositol phosphatidylcholine transfer protein (PITP). The expression of these genes was greatly reduced, up to 120,000 times, in the *rhl1.a* mutant in comparison to the ‘Karat’ variety. To answer the question of whether the genes encoding proteins identified in the present study as differentially accumulated were among the genes differentially expressed in transcriptome analysis, a qRT-PCR was used. For two genes corresponding to differentially accumulated proteins, a small but significant reduction of the expression between the mutant and the parent variety was found. For other genes, no significant differences in their expression between the wild-type/mutant pairs were noticed. Our bioinformatic analysis also showed that there are no probes on the microarrays for four genes corresponding to proteins identified as different in the proteomic study presented. This lack of correlation between the majority of information revealed by the transcriptome and proteome analysis in our studies may be explained by several factors.

First, there are many reports showing that data coming from transcriptome and proteome profiling are complementary to each other rather than being redundant, and numerous experiments have given an indication for the lack of complete consistency between these two sets of data. Several studies of transcriptomes and proteomes dealing with cell wall proteins (CWPs) and their corresponding genes from mature stems and dark-grown hypocotyl of *Arabidopsis* were reviewed by Pont-Lezica et al. (2010). These studies showed that many of the identified proteins originate from genes whose level of transcription was low or even below the background, which suggests a short half-life of the transcripts and low protein turnover (Pont-Lezica et al. 2010). In another study where transcriptome and proteome of rapeseed microspore-derived embryos were analysed, 14 transcript–protein pairs were selected based on a high sequence similarity between proteins and their corresponding translated cDNA probes. For the majority of such pairs, no correlation or only a weak correlation of their expression profiles was found. For three transcript–protein pairs from which corresponding cDNA probes were expressed in pollen, a negative correlation was noticed, indicating an opposite expression pattern at the mRNA and protein levels (Joosen et al. 2007). Similar conclusions were drawn from different studies of pollen transcriptome and proteome in *Arabidopsis* (reviewed by Becker and Feijó 2007) and rice (Dai et al. 2007), which strongly points to the complementarity of information derived from transcript and protein levels and the occurrence of multiple isoforms of proteins, which may originate from alternative splicing events. Another and extreme example of the discrepancy between transcriptome and proteome comes from the study performed on lung adenocarcinoma cancer by Chen et al. (2002), who found that only a small subset of the proteins exhibited a significant correlation with mRNA abundance. However, when the data for the level of all transcripts and proteins were compared, a negative correlation (*r* = −0.025) of their expression was obtained.

Conversely, when comparing the data from the proteomes of root hair mutants and their parent varieties with our previous transcriptome analysis, it is clear that no protein products of the genes reported by Kwasniewski et al. (2010) were identified as differentially accumulated in the present study. Such a finding is again supported by other literature data. Studies of CWPs and their corresponding genes (Pont-Lezica et al. 2010) showed that only around 11 % of genes detected in transcriptome studies were also found as their expression products at the proteome level. Most significantly, no AGPs were detected in proteome studies, which is related to the difficulties in their extraction from cell walls. Also, some proteins from the XET family were not detected, although their transcripts were present in the tissues studied. This may indicate that their expression regulation relies on some post-transcriptional events. The two above-mentioned protein classes showed similar patterns of identification in our studies—detection at the transcriptome level and the lack of detection at the proteome level.

The lack of the identification of proteins corresponding to other genes found as being differentially expressed in the root hairless mutant compared to the ‘Karat’ variety may result from similar reasons. The products of the majority of genes reported in our previous transcriptome study are localised to the cell walls and plasma membrane (Campbell and Braam 1999; Šamaj et al. 1999; Molendijk et al. 2001; Andrews et al. 2002; Seifert and Roberts 2007), which makes them less prone to extraction with the method used in the present study. Additional analysis performed using translated sequences of these genes and the Compute pI/Mw tool from the ExPASy portal (http://web.expasy.org/compute_pi/) showed that the theoretical pIs for two peroxidases, extensin and PITP, range from 7.93 to 8.98. Assuming no further modifications of these proteins, such a result indicates that they were out of the pH range of the IPG strips used in the presented study.

Taking all of the above into account, the differences observed between the root hair transcriptome and proteome analysis may result from either the technical limitations associated with the proteome analysis method applied in the study or from some biological events, such as the post-transcriptional regulation of gene expression, alternative splicing or differences in mRNA and protein longevity. Such results may also indicate that combining transcriptome and proteome analysis gives a more comprehensive view of the biological processes under study than is possible using only one of these methods alone.

It is worth mentioning that the majority of proteins or their homologues identified in the presented study were also identified in a root hair proteome analysis in maize (Nestler et al. 2011), with the exception of aluminium-induced protein-like protein, C2 domain-containing protein and PR17d precursor protein. However, eight other members of pathogen-related (PR) proteins were detected in the study of maize. As the proteome analysed by Nestler et al. (2011) came from fully developed, isolated root hairs, it can be assumed that similar proteins detected in our study are also characteristic for this type of root cells in barley. It also additionally proves that the comparison of mutants and their parent varieties is a good strategy for the detection of differentially accumulated proteins related to the mutated character.

In conclusion, it was possible to identify proteins that belong to several metabolic pathways and cellular functions. The identified factors participate in such processes as the control of vesicular trafficking, ROS signalling and homeostasis, signal transduction by the phospholipids metabolism and ATP transportation. Several proteins belong to families that have not previously been reported as being involved in root hair formation. Thus, the findings presented here may give novel information on the proteins important for root hair development in barley.

## Electronic supplementary material

Below is the link to the electronic supplementary material.

Table S1 Detailed list of the identified proteins, including the protein scores, peptide sequences, and scores and percentages of sequence coverage (XLS 53 kb)

## References

[CR1] Andrews J, Adams SR, Burton KS, Evered CE (2002). Subcellular localization of peroxidase in tomato fruit skin and the possible implications for the regulation of fruit growth. J Exp Bot.

[CR2] Arvidsson S, Kwasniewski M, Riaño-Pachón DM, Mueller-Roeber B (2008). QuantPrime—a flexible tool for reliable high-throughput primer design for quantitative PCR. BMC Bioinformatics.

[CR3] Becana M, Matamoros MA, Udvardi M, Dalton DA (2010). Recent insights into antioxidant defenses of legume root nodules. New Phytol.

[CR4] Becker JD, Feijó JA (2007). How many genes are needed to make a pollen tube? Lessons from transcriptomics. Ann Bot.

[CR5] Brechenmacher L, Lee J, Sachdev S, Song Z, Nguyen TH, Joshi T, Oehrle N, Libault M, Mooney B, Xu D, Cooper B, Stacey G (2009). Establishment of a protein reference map for soybean root hair cells. Plant Physiol.

[CR6] Campbell P, Braam J (1999). Xyloglucan endotransglycosylases: diversity of genes, enzymes and potential wall-modifying functions. Trends Plant Sci.

[CR7] Carol RJ, Dolan L (2006). The role of reactive oxygen species in cell growth: lessons from root hairs. J Exp Bot.

[CR8] Chen G, Gharib TG, Huang CC, Taylor JM, Misek DE, Kardia SL, Giordano TJ, Iannettoni MD, Orringer MB, Hanash SM, Beer DG (2002). Discordant protein and mRNA expression in lung adenocarcinomas. Mol Cell Proteomics.

[CR9] Christensen AB, Cho BH, Næsby M, Gregersen PL, Brandt J, Madriz-Ordeñana K, Collinge DB, Thordal-Christensen H (2002). The molecular characterization of two barley proteins establishes the novel PR-17 family of pathogenesis-related proteins. Mol Plant Pathol.

[CR10] Covitz PA, Smith LS, Long SR (1998). Expressed sequence tags from a root-hair-enriched *Medicago truncatula* cDNA library. Plant Physiol.

[CR11] Dai S, Chen T, Chong K, Xue Y, Liu S, Wang T (2007). Proteomics identification of differentially expressed proteins associated with pollen germination and tube growth reveals characteristics of germinated *Oryza sativa* pollen. Mol Cell Proteomics.

[CR12] Dash S, Van Hemert J, Hong L, Wise RP, Dickerson JA (2012). PLEXdb: gene expression resources for plants and plant pathogens. Nucleic Acids Res.

[CR13] Dowd PE, Coursol S, Skirpan AL, Kao TH, Gilroy S (2006). *Petunia* phospholipase c1 is involved in pollen tube growth. Plant Cell.

[CR14] Downes CP, Gray A, Lucocq JM (2005). Probing phosphoinositide functions in signaling and membrane trafficking. Trends Cell Biol.

[CR15] Dunwell JM, Khuri S, Gane PJ (2000). Microbial relatives of the seed storage proteins of higher plants: conservation of structure and diversification of function during evolution of the cupin superfamily. Microbiol Mol Biol Rev.

[CR16] Foreman J, Demidchik V, Bothwell JH, Mylona P, Miedema H, Torres MA, Linstead P, Costa S, Brownlee C, Jones JD, Davies JM, Dolan L (2003). Reactive oxygen species produced by NADPH oxidase regulate plant cell growth. Nature.

[CR17] Gahoonia TS, Nielsen NE (2004). Barley genotypes with long root hairs sustain high grain yields in low-P field. Plant Soil.

[CR18] Gilroy S, Jones DL (2000). Through form to function: root hair development and nutrient uptake. Trends Plant Sci.

[CR19] Guimil S, Dunand C (2007). Cell growth and differentiation in Arabidopsis epidermal cells. J Exp Bot.

[CR20] Hochholdinger F, Wen TJ, Zimmermann R, Chimot-Marolle P, da Costa e Silva O, Bruce W, Lamkey KR, Wienand U, Schnable PS (2008). The maize (*Zea mays* L.) *roothairless3* gene encodes a putative GPI-anchored, monocot-specific, COBRA-like protein that significantly affects grain yield. Plant J.

[CR21] Jones MA, Shen J-J, Fu Y, Li H, Yang Z, Grierson CS (2002). The Arabidopsis Rop2 GTPase is a positive regulator of both root hair initiation and tip growth. Plant Cell.

[CR22] Joosen R, Cordewener J, Supena ED, Vorst O, Lammers M, Maliepaard C, Zeilmaker T, Miki B, America T, Custers J, Boutilier K (2007). Combined transcriptome and proteome analysis identifies pathways and markers associated with the establishment of rapeseed microspore-derived embryo development. Plant Physiol.

[CR23] Kim WY, Cheong NE, Je DY, Kim MG, Lim CO, Bahk JD, Cho MJ, Lee SY (1997). The presence of a *Sar1* gene family in *Brassica campestris* that suppresses a yeast vesicular transport mutation *Sec12-1*. Plant Mol Biol.

[CR24] Kim SY, Sivaguru M, Stacey G (2006). Extracellular ATP in plants. Visualization, localization, and analysis of physiological significance in growth and signaling. Plant Physiol.

[CR25] Kwasniewski M, Szarejko I (2006). Molecular cloning and characterization of beta-expansin gene related to root hair formation in barley. Plant Physiol.

[CR26] Kwasniewski M, Janiak A, Mueller-Roeber B, Szarejko I (2010). Global analysis of the root hair morphogenesis transcriptome reveals new candidate genes involved in root hair formation in barley. J Plant Physiol.

[CR27] Libault M, Brechenmacher L, Cheng J, Xu D, Stacey G (2010). Root hair systems biology. Trends Plant Sci.

[CR28] Marchler-Bauer A, Lu S, Anderson JB, Chitsaz F, Derbyshire MK, DeWeese-Scott C, Fong JH, Geer LY, Geer RC, Gonzales NR, Gwadz M, Hurwitz DI, Jackson JD, Ke Z, Lanczycki CJ, Lu F, Marchler GH, Mullokandov M, Omelchenko MV, Robertson CL, Song JS, Thanki N, Yamashita RA, Zhang D, Zhang N, Zheng C, Bryant SH (2011). CDD: a Conserved Domain Database for the functional annotation of proteins. Nucleic Acids Res.

[CR29] Molendijk AJ, Bischoff F, Rajendrakumar CS, Friml J, Braun M, Gilroy S, Palme K (2001). *Arabidopsis thaliana* Rop GTPases are localized to tips of root hairs and control polar growth. EMBO J.

[CR30] Monteiro S, Piçarra-Pereira MA, Loureiro VB, Teixeira AR, Ferreira RB (2007). The diversity of pathogenesis-related proteins decreases during grape maturation. Phytochemistry.

[CR31] Nestler J, Schütz W, Hochholdinger F (2011). Conserved and unique features of the maize (*Zea mays* L.) root hair proteome. J Proteome Res.

[CR32] Nordberg J, Arnér ES (2001). Reactive oxygen species, antioxidants, and the mammalian thioredoxin system. Free Radic Biol Med.

[CR33] Padmanaban S, Lin X, Perera I, Kawamura Y, Sze H (2004). Differential expression of vacuolar H+-ATPase subunit c genes in tissues active in membrane trafficking and their roles in plant growth as revealed by RNAi. Plant Physiol.

[CR34] Panda SK, Baluška F, Matsumoto H (2009). Aluminum stress signaling in plants. Plant Signal Behav.

[CR35] Pfaffl MW, Horgan GW, Dempfle L (2002). Relative expression software tool (REST) for group-wise comparison and statistical analysis of relative expression results in real-time PCR. Nucleic Acids Res.

[CR36] Pont-Lezica R, Minic Z, Roujol D, San Clemente H, Jamet E, Osborne MA (2010). Plant cell wall functional genomics: novelties from proteomics. Advances in genetics research.

[CR37] Ramakers C, Ruijter JM, Deprez RH, Moorman AF (2003). Assumption-free analysis of quantitative real-time polymerase chain reaction (PCR) data. Neurosci Lett.

[CR38] Richards KD, Snowden KC, Gardner RC (1994). *wali6* and *wali7*. Genes induced by aluminum in wheat (*Triticum aestivum* L.) roots. Plant Physiol.

[CR39] Roux SJ, Steinebrunner I (2007). Extracellular ATP: an unexpected role as a signaler in plants. Trends Plant Sci.

[CR40] Šamaj J, Braun M, Baluška F, Ensikat H-J, Tsumuraya I, Volkmann D (1999). Specific localization of arabinogalactan-protein epitopes at the surface of maize root hairs. Plant Cell Physiol.

[CR41] Schiefelbein J, Kwak SH, Wieckowski Y, Barron C, Bruex A (2009). The gene regulatory network for root epidermal cell-type pattern formation in Arabidopsis. J Exp Bot.

[CR42] Seifert GJ, Roberts K (2007). The biology of arabinogalactan proteins. Annu Rev Plant Biol.

[CR43] Szarejko I, Janiak A, Chmielewska B, Nawrot M (2005). Genetic analysis of several root hair mutants of barley. Barley Genet Newsl.

[CR44] Wan J, Torres M, Ganapathy A, Thelen J, DaGue BB, Mooney B, Xu D, Stacey G (2005). Proteomic analysis of soybean root hairs after infection by *Bradyrhizobium japonicum*. Mol Plant Microbe Interact.

[CR45] Weerasinghe RR, Swanson SJ, Okada SF, Garrett MB, Kim SY, Stacey G, Boucher RC, Gilroy S, Jones AM (2009). Touch induces ATP release in Arabidopsis roots that is modulated by the heterotrimeric G-protein complex. FEBS Lett.

[CR46] Wen T-J, Schnable PS (1994). Analyses of mutants of three genes that influence root hair development in *Zea mays* (*Gramineae*) suggest that root hairs are dispensable. Am J Bot.

[CR47] Wen T-J, Hochholdinger F, Sauer M, Bruce W, Schnable PS (2005). The *roothairless1* gene of maize encodes a homolog of *sec3*, which is involved in polar exocytosis. Plant Physiol.

[CR48] Worrall D, Hird DL, Hodge R, Paul W, Draper J, Scott R (1992). Premature dissolution of the microsporocyte callose wall causes male sterility in transgenic tobacco. Plant Cell.

[CR49] Wu CT, Leubner-Metzger G, Meins F, Bradford KJ (2001). Class I {beta}-1,3-glucanase and chitinase are expressed in the micropylar endosperm of tomato seeds prior to radicle emergence. Plant Physiol.

[CR50] Xu J, Scheres B (2005). Dissection of Arabidopsis ADP-RIBOSYLATION FACTOR 1 function in epidermal cell polarity. Plant Cell.

[CR51] Yuo T, Toyota M, Ichii M, Taketa S (2009). Molecular cloning of a root hairless gene *rth1* in rice. Breed Sci.

[CR52] Zheng L, Shan J, Krishnamoorthi R, Wang X (2002). Activation of plant phospholipase Dβ by phosphatidylinositol 4,5-bisphosphate: characterization of binding site and mode of action. Biochemistry.

[CR53] Zimmermann G, Bäumlein H, Mock HP, Himmelbach A, Schweizer P (2006). The multigene family encoding germin-like proteins of barley. Regulation and function in basal host resistance. Plant Physiol.

